# Enhanced GIRK2 channel signaling in Down syndrome: A feasible role in the development of abnormal nascent neural circuits

**DOI:** 10.3389/fgene.2022.1006068

**Published:** 2022-09-12

**Authors:** Alexander M. Kleschevnikov

**Affiliations:** Department of Neurosciences, University of California San Diego, San Diego, CA, United States

**Keywords:** KCNJ6, early postnatal development, miR-155, depolarizing GABA, developmental GABA switch, neuronal excitability, gabaergic neurotransmission, synaptogenesis

## Abstract

The most distinctive feature of Down syndrome (DS) is moderate to severe cognitive impairment. Genetic, molecular, and neuronal mechanisms of this complex DS phenotype are currently under intensive investigation. It is becoming increasingly clear that the abnormalities arise from a combination of initial changes caused by triplication of genes on human chromosome 21 (HSA21) and later compensatory adaptations affecting multiple brain systems. Consequently, relatively mild initial cognitive deficits become pronounced with age. This pattern of changes suggests that one approach to improving cognitive function in DS is to target the earliest critical changes, the prevention of which can change the ‘trajectory’ of the brain development and reduce the destructive effects of the secondary alterations. Here, we review the experimental data on the role of *KCNJ6* in DS-specific brain abnormalities, focusing on a putative role of this gene in the development of abnormal neural circuits in the hippocampus of genetic mouse models of DS. It is suggested that the prevention of these early abnormalities with pharmacological or genetic means can ameliorate cognitive impairment in DS.

## Introduction

Down syndrome (DS), a genetic disorder caused by the triplication of genes on the human chromosome 21 (HSA21) (Lejeune et al., 1959), is characterized by moderate to severe cognitive impairment. The mechanisms underlying to cognitive impairment in DS are being methodically investigated (Lott, 2012; Contestabile et al., 2017; Rafii et al., 2019; Antonarakis et al., 2020; Botte and Potier, 2020). The abnormalities can be conditionally divided into two groups: primary changes, caused directly by overexpression of the HSA21 genes, and secondary changes, arising mainly as compensatory reactions to abnormal development (Sturgeon et al., 2012; Antonarakis, 2017; Hasina et al., 2022). Due to the accumulation of these secondary abnormalities, initially mild developmental deficits become severe with age. This suggests that one approach to improving cognitive function in DS is to target the early primary changes, which can reduce or prevent the development and, thus, the destructive effects of the secondary alterations. In this review, we focus on the role of the *KCNJ6* gene in DS-specific brain abnormalities. In particular, we will discuss the putative role of *KCNJ6* in the development of abnormal neural circuits in genetic mouse models of DS. It is suggested that prevention of these early abnormalities through pharmacological or genetic means may improve cognitive impairment in DS.

## Role of KCNJ6 in Down syndrome and other developmental disorders

*KCNJ6* is located in the middle of the “Down syndrome critical region” (DSCR) of HSA21, the triplication of which is necessary for the manifestation of cognitive impairment (McCormick et al., 1989; Korenberg et al., 1990; Peterson et al., 1994), and is sufficient in mouse genetic models to confer behavioral, neurophysiological, and synaptic phenotypes characteristic of DS (Belichenko N. P. et al., 2009; Jiang et al., 2015). Recent reanalysis of all ‘partial trisomy 21’ cases described from 1973 to 2021 revealed an extremely narrow (34 kbp) subregion of DSCR, most closely related to the DS diagnosis (Pelleri et al., 2016; Pelleri et al., 2019; Antonaros et al., 2021). Only two genes, *DSCR4* and *KCNJ6*, span across this subregion. *DSCR4* is a *de novo*-originated protein-coding gene that is likely absent in mice (Toyoda et al., 2002). In people, it is predominantly expressed in the placenta (Asai et al., 2008) and is involved in the regulation of biological pathways related to cell migration, coagulation, and the immune system (Saber et al., 2021). The role of *DSCR4* in cognition is unknown. In contrast, *KCNJ6* has been implicated in several genetic abnormalities characterized by cognitive impairment, as well as in the modulation of a variety of higher brain functions. Thus, heterozygous mutations of *KCNJ6,* affecting the pore-forming domain of the GIRK2 channel, result in the Keppen-Lubinsky syndrome, a genetic disorder characterized by severe developmental delay, microcephaly, and intellectual disability (Basel-Vanagaite et al., 2009; Masotti et al., 2015). Genetic variations of *KCNJ6* were implicated in the modulation of reward-related brain processes (Kamarajan et al., 2017; Ziegler et al., 2020) and pain sensitivity (Nishizawa et al., 2014; Langford et al., 2015; Elens et al., 2016). *KCNJ6* may also play an important role in the generation of infantile spasms associated with cognitive impairment (Cortez et al., 2009; Blichowski et al., 2015; Joshi et al., 2016), as well as in other disorders (Mayfield et al., 2015; Jeremic et al., 2021). In rodents, a missense mutation of *KCNJ6* is responsible for the ‘weaver’ phenotype, characterized by abnormal development of the cerebellum, ectopic cells in the CA3 region, and other developmental abnormalities (Patil et al., 1995; Sekiguchi et al., 1995; Ozaki et al., 2002; Schein et al., 2005). Overexpression of *KCNJ6* impairs synaptic plasticity and provokes cognitive impairment resembling DS in wild type mice (Cooper et al., 2012). Thus, *KCNJ6* abnormalities may influence brain development and lead to cognitive impairment, supporting the notion that triplication of this gene plays an important role in cognitive impairment in DS.

## Properties of GIRK2 channels

The *KCNJ6* gene encodes GIRK2 subunits of G protein-activated inwardly rectifying potassium channels. Alternative splicing of *KNCJ6* results in the generation of four GIRK2 isoforms (GIRK2a, GIRK2b, GIRK2c, and GIRK2d), which differ in trafficking and therefore in the regional and subcellular distribution (Wei et al., 1998; Harashima et al., 2006b; Marron Fernandez de Velasco et al., 2017). Functional potassium channels are formed by GIRK2 as heterotetramers in combination with other subunits (GIRK1, GIRK3, and GIRK4) or as homotetramers (Yamada et al., 1998; Inanobe et al., 1999; Hibino et al., 2010). GIRK2 channels serve as effectors for a number of postsynaptic metabotropic receptors such as GABAB, A1, 5-HT1A, m2, and others (Luscher et al., 1997; Yamada et al., 1998; Dascal and Kahanovitch, 2015). In addition, GIRK2 channels are activated constitutively in an agonist-independent manner (Chen and Johnston, 2005; Kim and Johnston, 2015). High-resolution immunohistochemical studies showed that, in the hippocampus, GIRK2 channels are most abundant at the perisynaptic locations on dendritic shafts and spines of principle neurons, where they co-localized with GABAB receptors (Kulik et al., 2006). Activation of GIKR2 channels requires simultaneous action of beta-gamma subunits of G-proteins, which are produced following activation of the corresponding G protein-coupled metabotropic receptors, and PIP2 (Whorton and MacKinnon, 2011; Wang et al., 2014). Since GIRK2 channels are selective for K+ ions, opening of these channels shifts the membrane potential in the direction of equilibrium potential for potassium (∼ -94 mV), thus hyperpolarizing neurons. Both the hyperpolarization and the reduction of membrane resistance upon activation of these channels reduces neuronal excitability. Thus, GIRK2 channels play an important role in the maintenance of excitatory/inhibitory balance by effectively controlling the resting membrane potential and neuronal excitability.

## GIRK2 channels in Down syndrome models

Mouse genetic models have been successfully used to examine structural and functional abnormalities of DS in invasive experiments (Dierssen et al., 2001; Belichenko et al., 2004; Salehi et al., 2006; Yu et al., 2010; Gotti et al., 2011; Liu et al., 2011; Popov et al., 2011; Kleschevnikov et al., 2012c; Cramer and Galdzicki, 2012; Haydar and Reeves, 2012; Ruparelia et al., 2012; Zhang et al., 2012; Zhang et al., 2014; Belichenko et al., 2015). In addition, genetic models of DS have been instrumental for the discovery and evaluation of potential treatments to improve the impaired cognition (Dierssen et al., 2001; Belichenko et al., 2007; Dierssen et al., 2009; Salehi et al., 2009; Faizi et al., 2011; Kleschevnikov et al., 2012a; Kleschevnikov et al., 2012c; Haydar and Reeves, 2012; Lott, 2012; Mohler, 2012; Rueda et al., 2012; Ruparelia et al., 2012; Lysenko et al., 2014; Martinez-Cue et al., 2014; Belichenko et al., 2016; Hamlett et al., 2016; Reeves et al., 2019; Tayebati et al., 2019; Chen et al., 2020). The most extensively used genetic model of DS, Ts65Dn mice, are segmentally trisomic for the mouse chromosome 16 region that is syntenic to a subregion of HSA21 containing DSCR. These mice exhibit multiple structural, physiological, and behavioral phenotypes characteristic of DS (Freeburn and Munn, 2021).

In Ts65Dn mice, levels of Girk2 are increased by about 50% in the hippocampus, neocortex, and other brain regions, consistent with the increased dose of *KCNJ6* (Kleschevnikov et al., 2005; Harashima et al., 2006b; Kleschevnikov et al., 2012b). The expression of all splicing GIRK2 isoforms is increased (Harashima et al., 2006a). Regional distribution of GIRK2 is also altered with a disproportional increase of GIRK2 levels in the outer molecular layer of the dentate gyrus (DG) and the lacunosum moleculare of the CA3 (Harashima et al., 2006a). The targeting of GIRK2 channels to the cellular membrane is also enhanced in DS (Wang et al., 2013). This effect is mediated by an increased expression of *miR-155*, which reduces expression levels of sorting nexin 27, thereby reducing trafficking of GIRK2 from the cellular membrane to endosomes (Wang et al., 2013).

Increased membrane targeting and expression of GIRK2 channels implies that their efficiency is enhanced in DS models. Thus, the selective GABAB receptor agonist baclofen evoked larger whole-cell currents and caused a greater reduction of the input resistance thus signifying that GABAB/GIRK2 signaling is enhanced in cultured hippocampal neurons of Ts65Dn mice (Best et al., 2007). Baclofen-evoked currents were also increased, and the resting membrane potential of the DG granule neurons was excessively hyperpolarized, in acute hippocampal slices from Ts65Dn mice (Kleschevnikov et al., 2012b). These observations show that GIRK2 signaling is significantly enhanced thus contributing to the increased overall inhibitory efficiency in genetic models of DS.

## Downregulation of GIRK2 channels improves synaptic plasticity and cognition in adult Ts65Dn mice

The increased GABAB/GIRK2 signaling can reduce synaptic plasticity and thus lead to deficient cognition in DS models. If so, downregulating GABAB/GIRK2 currents can improve both synaptic plasticity and cognition. This can be achieved either pharmacologically with GABAB receptor antagonists or GIRK2 channel blockers, or genetically by reducing *KCNJ6* gene dose. Thus, long-term potentiation in the dentate gyrus (Kleschevnikov et al., 2012a) and perirhinal cortex (Roncace et al., 2017) of Ts65Dn mice was improved by selective GABAB receptor antagonists. Direct blocking of GIRK2 channels with fluoxetine, a serotonin reuptake inhibitor that effectively suppresses GIRK2 currents (Kobayashi et al., 2003), was also effective in the restoration of long-term potentiation (LTP) in Ts656Dn mice (Kleschevnikov et al., 2017). This action of fluoxetine cannot be attributed to an increase in serotonin level since this would suppress LTP in the dentate gyrus (Sakai and Tanaka, 1993) or to a promotion of adult neurogenesis (Clark et al., 2006) since this would require a longer amount of time. Ethosuximide, another partial blocker of GIRK2 channels (Kobayashi et al., 2009), was not effective in terms of cognitive recovery or other improvements in adult Ts65Dn mice (Vidal et al., 2012). However, this drug also inhibits low-threshold T-type Ca+2 channels, which may negatively affect synaptic plasticity and memory (Ponnusamy and Pradhan, 2006; Leresche and Lambert, 2017), thus mitigating the possible positive effects of GIRK2 channel blockade. Finally, LTP was restored in Ts65Dn mice that only had two functional copies of *KCNJ6* (Kleschevnikov et al., 2017). All these treatments also improved the cognitive function in Ts65Dn mice. Chronic i. p. injections of the GABAB antagonist CGP55845 restored long-term memory in the Novel Object Recognition and Contextual Fear Conditioning tests (Kleschevnikov et al., 2012a). Interestingly, not all abnormal behavioral parameters were restored by this treatment. For example, abnormal working memory and increased locomotor activity were not affected (Kleschevnikov et al., 2012a). Similar to GABAB antagonists, chronic administration of fluoxetine also improved spatial memory in adult Ts65Dn mice (Begenisic et al., 2014). However, no improvement of cognition was observed in another study in which an excessively high dose of fluoxetine had been used (Heinen et al., 2012). These results indicate that the suppression of GABAB/GIRK2 signaling must be within certain limits to be effective. In addition, since fluoxetine increases the rate of adult neurogenesis mice (Clark et al., 2006), it is not clear to what degree the suppression of GIRK2 channels was responsible for the cognitive improvement in this study. Finally, restoration of long-term memory was observed in Ts65Dn mice that had only two copies of *KCNJ6* (Kleschevnikov et al., 2017). Thus, moderate suppression of GIRK2 channels restores hippocampal synaptic plasticity and long-term memory in Ts65Dn model of DS. On the other hand, many aspects of abnormal behavior do not improve with such treatments. It is therefore important to understand how and when those abnormalities arise during the brain development in DS models.

## Formation of nascent neuronal circuits

**FIGURE 1:**
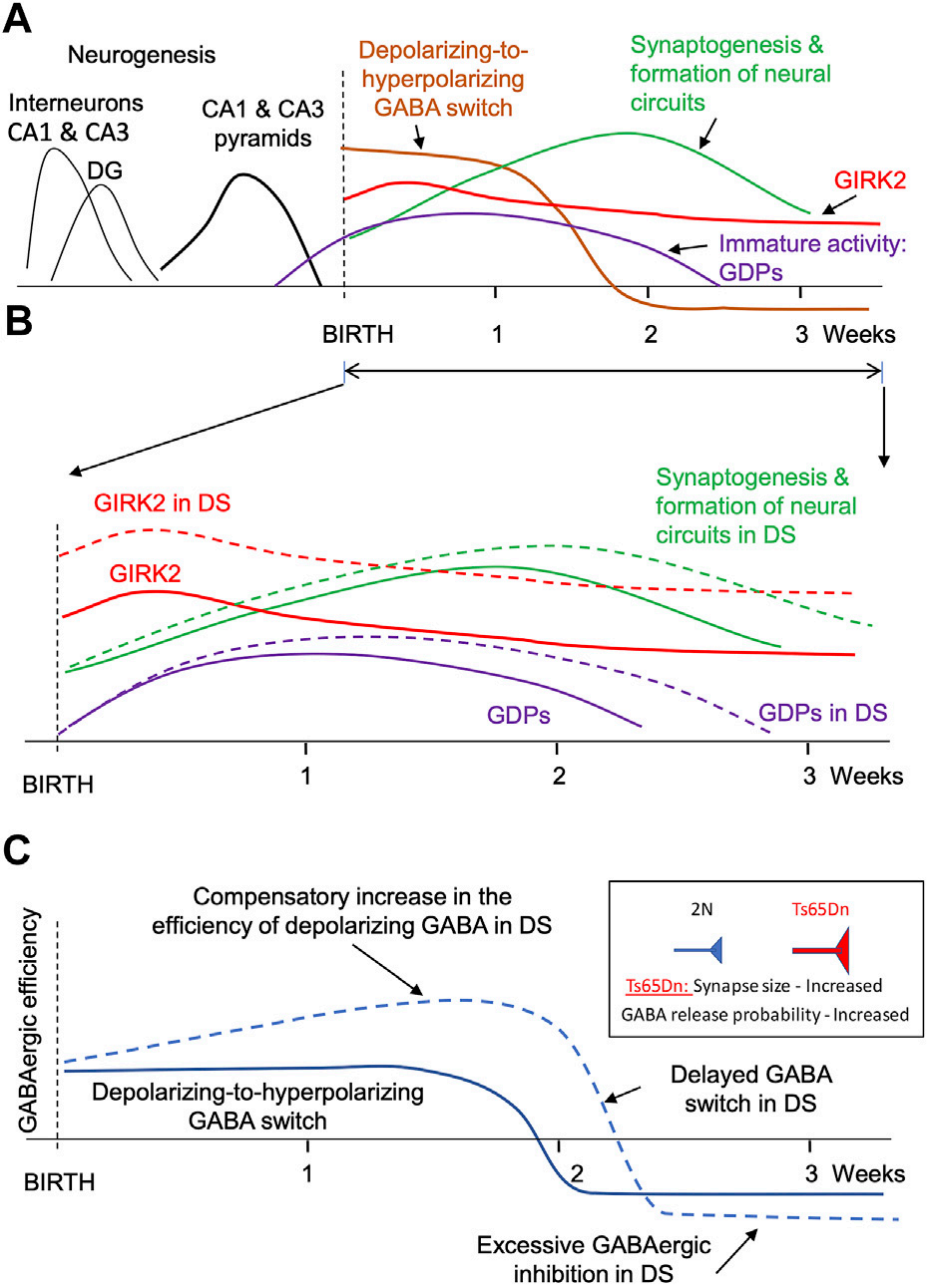
Major events during the formation of faulty neuronal circuits in the hippocampus of mouse genetic model of DS. **(A)**. Main events in the hippocampus of normosomic mice. The time course of neurogenesis (black lines), synaptogenesis (green), GIRK2 expression (red), depolarizing-to-hyperpolarizing GABA switch (brown), and immature giant depolarizing potentials (GDP, purple). **(B)**. Changes in GIRK2 expression levels (red), immature activity (violet), and neural circuit formation (green). Dashed lines show the changes in DS mice. Note the increase in GIRK2 levels and the prolongation of the period of immature activity in DS models. **(C)**. Putative changes in the efficiency of GABAergic neurotransmission in the hippocampus of mouse genetic models during early postnatal development. Note a compensatory elevation of the depolarizing GABAergic efficiency before the GABA switch. The increased GABAergic excitation turns into an increased GABAergic inhibition after the GABA switch.

In rodents, the formation of neural circuits starts in the prenatal period and continues postnatally for several weeks. In the CA1 and CA3 regions, neurogenesis is mostly completed before birth (Figure 1A, black lines). However, astrogenesis and synaptogenesis last for several postnatal weeks (Figure 1A, green line). The formation of correct synaptic connections during this period critically depends on spontaneous neuronal activity (Kirkby et al., 2013; Kerschensteiner, 2014; Luhmann et al., 2016; Griguoli and Cherubini, 2017). Factors that affect neuronal excitability and neuronal firing can thus interfere with the formation of neural circuits.

One factor affecting excitability and spontaneous activity of neonatal neurons is the depolarizing action of GABA. Thus, in early development GABA depolarizes neurons due to high intracellular chloride level (Rivera et al., 1999; Ben-Ari et al., 2007) (Figure 1A, brown line). Because glutamatergic synapses are not yet fully developed at this time (Tyzio et al., 1999), neuronal depolarization caused by the activation of GABAA receptors provides an important source of neuronal excitation (Rivera et al., 1999; Ben-Ari, 2002; Blankenship and Feller, 2010). Later in development, after the formation of glutamatergic synapses, the polarity of GABA action switches from depolarizing to hyperpolarizing (Ben-Ari et al., 2007) (Figure 1A, brown line). Therefore, changes in the duration or strength of the depolarizing GABA action in early development lead to the formation of abnormal dendrites, synaptic connections, faulty neural circuits (Cancedda et al., 2007; Levav et al., 2008; Levav-Rabkin et al., 2010; Chen and Kriegstein, 2015), and abnormal behavior in adulthood (Levav et al., 2004; Melamed et al., 2014).

Activation of GIRK2 channels represents another important factor affecting neuronal excitability and hence spontaneous activity. GIRK2 is expressed at high levels during the perinatal and early postnatal periods (Karschin and Karschin, 1997; Fernandez-Alacid et al., 2011; Aguado et al., 2013). In the cerebellum, GIRK2 levels peak at P5 and gradually decrease over several weeks to adult levels that are 3 times lower than in the early postnatal period (Aguado et al., 2013). GIRK2 levels are also high in the neocortex and hippocampus early in the postnatal period (Fernandez-Alacid et al., 2011) (Figure 1A, red line). High expression levels of GIRK2 during the perinatal and early postnatal periods suggest that altered signaling through these channels can interfere with the formation of nascent neural circuits in DS.

## Early neuronal abnormalities in Down syndrome: Possible role of GIRK2 channels

The earliest brain abnormalities in DS are observed in prenatal development and include changes in the expression of proteins (Engidawork and Lubec, 2003; Lott, 2012), reduced neurogenesis (Sylvester, 1983), and disorganized cortical lamination (Golden and Hyman, 1994; Utagawa et al., 2022). However, the most noticeable physiological and morphological alterations occur after birth, when dendritic and synaptic abnormalities quickly accumulate in the cerebral cortex and hippocampus (Marin-Padilla, 1976; Takashima et al., 1989; Schmidt-Sidor et al., 1990; Engidawork and Lubec, 2003; Aldridge et al., 2007). At this period, GIRK2 is already expressed at high levels in the brain of DS individuals (Thiery et al., 2003). In genetic mouse models, the first abnormalities are also observed in the prenatal period (Haydar et al., 1996; Haydar et al., 2000; Cheng et al., 2004; Chakrabarti et al., 2007; Haydar and Reeves, 2012). In Ts65Dn mice, delays in growth of the neocortex and hippocampus have been noted with the first changes seen on E14.5 (Chakrabarti et al., 2007). However, like in people with DS, the most significant alterations have been observed in postnatal (P0-P16) development (Lorenzi and Reeves, 2006; Contestabile et al., 2007; Lysenko et al., 2018; Jain et al., 2020; Uguagliati et al., 2022). These postnatal changes were accompanied by significant delays in achieving developmental motor and sensory milestones (Holtzman et al., 1996; Toso et al., 2008; Olmos-Serrano et al., 2016).

Rapid accumulation of the DS-specific brain abnormalities in the early postnatal period suggests that some of these changes could be compensatory, caused by the changes in the expression of the genes present on HSA21. It is therefore feasible that these secondary changes can be mitigated by time-limited pharmacological or genetic interventions at the time or soon after their origin. One possible scenario could be the that an increased expression of *KCNJ6* and *miR-155*, both on HSA21, increases the efficiency of the GIRK2 channel signaling (the primary change) (Figure 1B, red dashed line). This provokes excessive hyperpolarization and reduced excitability of neonatal neurons, which reduces their spontaneous activity (Madamba et al., 2021). Spontaneous activity plays an important role in synaptogenesis and in establishing correct synaptic connections (Kirkby et al., 2013; Kerschensteiner, 2014). Thus, a decrease in neonatal spontaneous activity will delay and disrupt the formation of neural circuits (Figure 1B, green dashed). In confirmation of this, a prolongation of the period of immature neuronal activity such as generation of the giant depolarizing potentials (GDPs) was observed in neonatal Ts65Dn mice (Lysenko et al., 2018) (Figure 1B, violet dashed line).

An additional outcome of the reduced neonatal spontaneous activity could be an increase in the efficiency of GABAergic neurotransmission in Ts65Dn mice (Figure 1C). Indeed, the depolarizing GABA action is one of the main sources of neonatal neuronal activation. A decrease in spontaneous activity usually triggers homeostatic compensatory changes to restore this activity (Turrigiano and Nelson, 2004; Wefelmeyer et al., 2016; Tien and Kerschensteiner, 2018). One such change may be an increase in the efficiency of depolarizing GABA (Figure 1C, upper part of the blue dashed line). For example, a decrease in spontaneous network activity causes an increase in the amplitude of the excitatory GABAergic postsynaptic currents in neonatal spinal motoneuron inputs (Gonzalez-Islas and Wenner, 2006). After switching the GABA action from depolarizing to hyperpolarizing, this change results in an increased GABAergic inhibition (Figure 1C, bottom part of the blue dashed line). The efficiency of GABAergic neurotransmission is increased in Ts65Dn and other genetic models of DS (Kleschevnikov et al., 2004; Belichenko P. V. et al., 2009; Kleschevnikov et al., 2012b; Kleschevnikov et al., 2012c). Interestingly, the increase in GABAergic efficiency is associated with an increase in the size of GABAergic synapses (Belichenko P. V. et al., 2009) and the presynaptic release probability of GABA (Kleschevnikov et al., 2004; Kleschevnikov et al., 2012b), which are characteristic of compensatory changes caused by a decrease in neuronal activity (Murthy et al., 2001; Han and Stevens, 2009).

Provided that this scenario is correct, one approach to improve cognitive impairment in DS is to reduce GIRK2 channel signaling during the formation of the nascent neural circuits. An indirect confirmation of the validity of such approach is the improvement of synaptic plasticity and cognition in Ts65Dn mice following prenatal (Guidi et al., 2014) and neonatal (Bianchi et al., 2010; Stagni et al., 2015) treatment with fluoxetine, which, among other effects, suppresses GIRK2 channels. However, since fluoxetine has many other effects, it is not clear if and whether some of the improvements were due to the suppression of GIRK2 channels in these studies. Additional investigations are needed to either prove or disprove the critical role of GIRK2 channel signaling in the formation of abnormal neural circuits in DS.

## Conclusion: Methodology of cognitive correction in DS

Theoretically, two major strategies can be used to improve cognitive function in DS. The first strategy is to correct genetic abnormalities, on the assumption that this will eventually lead to the correction of various brain functions, including cognition. Several attempts have been made to implement this strategy. For example, silencing of genes on one chromosome 21 using the integrated XIST (X-Inactivation Specific Transcript) transgene broadly repressed this chromosome in DS pluripotent cells, partially correcting cell pathogenesis in an *in vitro* model of human fetal hematopoiesis (Li et al., 2012; Jiang et al., 2013; Plona et al., 2016; Chiang et al., 2018). In other studies, the corrections were restricted to one or several genes of interest. e.g., correction of the gene dose of *Dyrk1a* (Altafaj et al., 2013; Garcia-Cerro et al., 2017), *Kcnj6* (Kleschevnikov et al., 2017), or *App* (Salehi et al., 2006; Mojabi et al., 2016) improved neuronal properties and behavior in mouse models of DS. The second strategy is to identify critical synaptic and cellular abnormalities leading to impaired brain development and then directly correct these abnormalities with pharmacological, genetic, or other means. This can be achieved not only in mouse genetic models but also in humans by comparing the development of cell lines or organoids derived from isogenic pairs of trisomy 21 and euploid induced pluripotent stem cells (iPSCs) (Weick et al., 2016; Araujo et al., 2018; Real et al., 2018; Kawatani et al., 2021; Tang et al., 2021; Nehra et al., 2022; Sharma et al., 2022; Xu et al., 2022). It is important to note that this second strategy does not require correction of all genetic abnormalities, and it does not target all DS-specific phenotypes. While implementing such strategy, it was previously shown that the efficiency of GABAergic inhibition is increased in the hippocampus of DS mice, resulting in decreased synaptic plasticity (Kleschevnikov et al., 2004). Correction of the increased inhibition with antagonists of GABAA (Kleschevnikov et al., 2004; Fernandez et al., 2007), GABAB (Kleschevnikov et al., 2012a), or inverse agonists of α5 subunit-containing GABAA receptors (Braudeau et al., 2011; Martinez-Cue et al., 2013) improved both synaptic plasticity and cognition in mouse genetic models of DS.

Although the first strategy looks preferable, it has several limitations restricting its practical use. Thus, not all genes on chromosome 21 could be suppressed by the XIST transgene or other methods, leaving many genes expressed at higher levels. This problem is especially noticeable in the case of correction of only one or several triplicated genes. Next, expression changes in DS are genome-wide and cannot be corrected by silencing of genes only on chromosome 21. Most importantly, such genetic therapy requires germline genetic alterations, which is highly controversial and currently ethically precluded. These limitations are absent in the second strategy.

In our view, the most promising approach is a combination of these two strategies. As described in this review, *KCNJ6* gene was identified a critical target for genetic correction in DS, because: 1) Increased expression of *KCNJ6* alone results in phenotypes reminiscent of DS (Cooper et al., 2012), and 2) Correction of *KCNJ6* dose restores synaptic plasticity and cognition in the Ts65Dn model (Kleschevnikov et al., 2017). This change likely affects neonatal neuronal activity, thus resulting in the development of abnormal synaptic circuits. We can thus hypothesize that correcting these critical abnormalities in the neonatal brain by time-limited pharmacological or genetic interventions may normalize the development of nascent neural circuits and thereby improve cognitive function in DS. These expectations should be tested in the future studies.
